# Protection against oxidative stress mediated by the Nrf2/Keap1 axis is impaired in Primary Biliary Cholangitis

**DOI:** 10.1038/srep44769

**Published:** 2017-03-23

**Authors:** Urszula Wasik, Małgorzata Milkiewicz, Agnieszka Kempinska-Podhorodecka, Piotr Milkiewicz

**Affiliations:** 1 Department of Medical Biology Laboratory, Pomeranian Medical University, Szczecin, Poland; 2 Liver and Internal Medicine Unit, Medical University of Warsaw, Warsaw, Poland; 3 Translational Medicine Group, Pomeranian Medical University, Szczecin, Poland

## Abstract

In response to oxidative stress, nuclear factor (erythroid-derived 2)-like2 (Nrf2) induces expression of cytoprotective genes. The Nrf2 pathway is controlled by microRNAs and Kelch-like ECH-associated protein1 (Keap1). *Nrf2 is stabilized when Keap1 is* degraded through the autophagy pathway in a p62-dependent manner. The inhibition of autophagy causes protein accumulation, and Keap1 is inactivated by binding to p62. We investigated the role of the Nrf2/Keap1 axis in the amelioration of oxidative stress in primary biliary cholangitis (PBC). Liver specimens from patients with PBC, with (n = 24) or without cirrhosis (n = 14), and from controls (n = 16) were used for molecular analyses. We found that Nrf2 protein levels were elevated in PBC compared to controls, but Nrf2 gene expression was significantly reduced in cirrhotic PBC. Nrf2 target gene products, HO-1 and GCLC proteins, were reduced compared to controls and reduction of Nrf2 gene expression was associated with elevated levels of microRNA-132 and microRNA-34a. Both Keap1 and p62 protein levels were substantially increased in PBC compared to controls. PBC was associated with reduced Nrf2 expression and autophagy deterioration and these impairments were more advanced in patients with cirrhosis. Aberrant Nrf2/Keap1 system integrity may affect self-defence mechanisms against oxidative stress in PBC.

Primary biliary cholangitis (PBC)^1^ is a chronic, progressive cholestatic liver disease that typically affects middle-aged women and may lead to liver failure and transplantation^2^. PBC is considered to have an autoimmune aetiology, because highly specific anti-mitochondrial antibodies are present in nearly 95% of patients. The abnormal immune response during PBC development leads to the activation and expansion of autoreactive T and B lymphocytes, followed by the production of numerous inflammatory mediators^3^,^4^,^5^. As a result, the liver is subjected to a cascade of destructive events, including oxidative stress. When serum samples from patients with PBC were screened with mass spectrometry, the results revealed alterations in redox homeostasis characterized by elevated levels of biochemical markers of oxidative stress, including lipid peroxidation and cholesterol auto-oxidation^6^. The presence of oxidative stress-related metabolites contributed to an enhanced immune response. Hence, patients with PBC have high levels of IgGs against the adduct of malondialdehyde, the most important product of lipid peroxidation, and against human serum albumin^7^. In addition to altering lipid composition, oxidative stress in PBC leads to accumulations of protein oxidative products and alterations in glutathione metabolism^8^. UDCA treatment of PBC seems to improve redox changes in serum and liver tissues^9^,^10^,^11^,^12^.

Nuclear factor-E2-related factor 2 (Nrf2) is a short-lived protein that acts as a transcription factor. Nrf2 drives the expression of numerous cytoprotective genes involved in xenobiotic metabolism, antioxidant responses, and anti-inflammatory responses. Proteins upregulated by Nrf2 signalling include haeme oxygenase-1 (HO-1), superoxide dismutase (SOD1), catalase, and enzymes involved in glutathione metabolism, such as glutathione S-transferase, glutathione cysteine ligase modifier subunit, and glutathione cysteine ligase catalytic subunit (GCLC)^13^,^14^,^15^,^16^. Activation of Nrf2 signalling is fine-tuned by the actin-associated kelch-domain 1 protein (Keap1), an adaptor component of Cullin 3 (Cul3)-base ubiquitin E3 ligase. Keap1 either sequesters Nrf2 in the cytoplasm or facilitates Nrf2 ubiquitination and degradation. The integrity of the Nrf2/Keap1 system depends on a multifaceted protein, known as p62 (also, SQSTM1, Sequestosome1, A170). p62 regulates cellular redox homeostasis by binding to Keap1 and facilitating its degradation in an autophagy-dependent manner^17^. Consequently, Nrf2 accumulates, and then, translocates to the nucleus, where it targets a cis-acting antioxidant response element, which activates a set of genes. In addition to p62, the Nrf2/Keap1 signalling axis is regulated by epigenetic mechanisms that involve microRNAs^18^,^19^. To date, numerous microRNAs have been identified that function as modulators of Nrf2 and related regulatory proteins^20^,^21^.

Dysregulation of the Nrf2 pathway is implicated in various diseases, including lung, ovarian, prostate, and breast cancers; and in inflammatory conditions, including hepatitis, diabetes, atherosclerosis, and neurodegenerative disease^22^,^23^,^24^,^25^. Despite the fact that PBC progression manifests with features of oxidative stress, data are lacking about Nrf2 signalling, a key player in antioxidant defence. Therefore, this study investigated the interplay between Nrf2 and Keap1 in PBC.

## Results

### In PBC, Nrf2 protein levels are elevated, but mRNA levels are diminished

**Figure 1:**
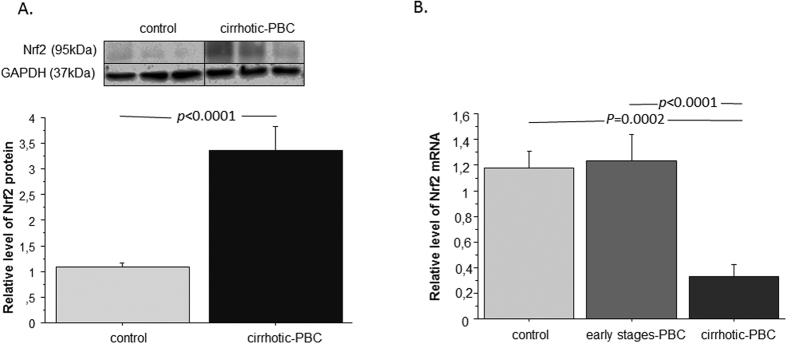
Nrf2 expression in liver tissues of patients with PBC and controls. **(A**) Nrf2 protein expression was increased in liver tissue of patients with PBC in comparison to controls Protein levels were determined by densitometry after normalization to GAPDH as a control for loading. Images are representative of at least six experiments. (**B**) The level of Nrf2 mRNA, estimated by real-time PCR, was decreased in liver tissue of patients with cirrhotic PBC but not in early-stages PBC patients when compared to controls. The results were normalized to 18sRNA. Bars indicate the mean ± SEM.

We analysed Nrf2 mRNA and Nrf2 protein levels in patients with PBC and controls (Fig. 1). Nrf2 protein levels were significantly increased in patients with PBC compared to controls (3.3-fold; p < 0.0001; Fig. 1A). Simultaneously, Nrf2 mRNA levels were 3.5-fold lower in patients with PBC and cirrhosis, compared to controls (p = 0.0002) and patients in the early stages of PBC (p < 0.0001; Fig. 1B).

### Nrf2 target genes are downregulated in PBC

**Figure 2:**
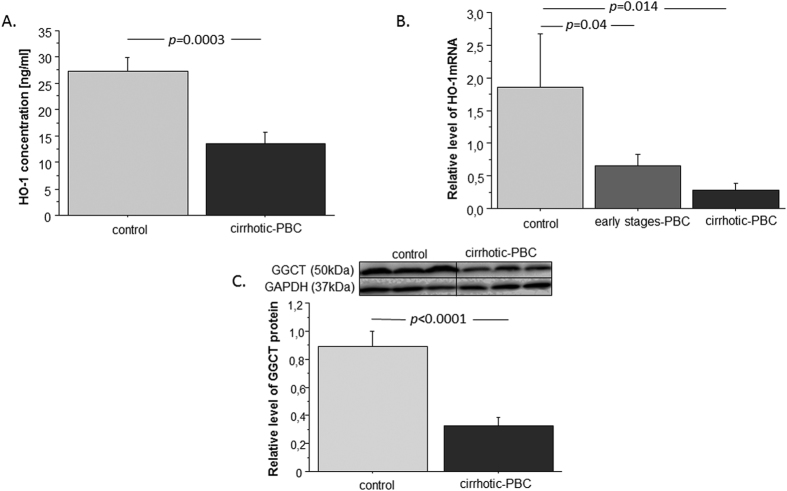
Expression of HO-1 and GCLC in liver tissues of patients with PBC and controls. (**A**) Concentration of HO-1 protein was lower within PBC livers in comparison to controls. (**B**) HO-1 mRNA level, estimated by real-time PCR, was decreased in liver tissue of both cirrhotic PBC and early-stages PBC when compared to controls. The results were normalized to 18sRNA. (**C**) GCLC protein levels were assessed in liver tissue of cirrhotic PBC and controls and quantified relative to GAPDH. Bars indicate the mean ± SEM.

To assess the antioxidative response of Nrf2 in PBC, we estimated its induction of the downstream target genes, *HO-1* and *GCLC* (Fig. 2.). First, we checked HO-1 protein concentrations in liver extracts with ELISAs (Fig. 2A). We found that HO-1 levels were 2-fold lower in patients with PBC than in the control group (p = 0.0003). Moreover, HO-1 mRNA expression levels were 9-fold lower in patients with PBC than in controls (p = 0.014; Fig. 2B). Patients in the early stages of PBC also had 2.8-fold lower HO-1 expression than controls (p = 0.04). Among patients with PBC, there was no significant difference in HO-1 expression between those with cirrhosis and those in the early stages of the disease. Next, we estimated the Nrf2 induction of GCLC gene expression. We checked GCLC protein levels with Western blot analyses (Fig. 2C). We found that patients with PBC had 2.7-fold lower GCLC levels than controls (p < 0.0001).

### MicroRNAs, miR-132 and miR-34a, showed enhanced expression in PBC livers

**Figure 3:**
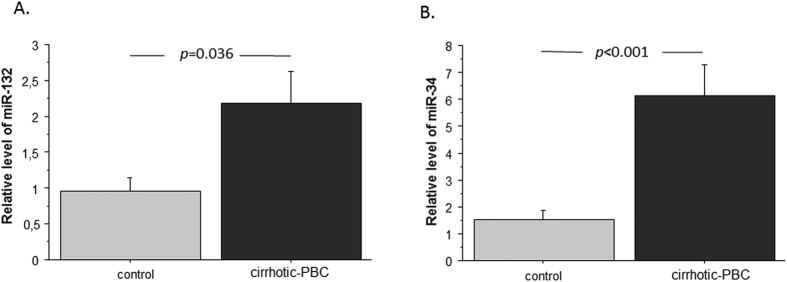
Expression of miR-132 and miR-34a microRNAs in liver tissues of patients with PBC and controls. Changes in (**A**) miR-132 and (**B**) miR-34a levels, determined with real-time PCR. The results were normalized to miR-191. Bars indicate the mean ± SEM.

Nrf2 gene expression is driven by numerous factors, including microRNAs. We analysed microRNA expression with Affymetrix GeneChip miRNA 4.0 arrays. The results revealed that miR-132 and miR-34a were overexpressed in livers of patients with PBC by 2.3-fold, (*p* = 0.001) and 5-fold (*p* < 0.0001), respectively, compared to control livers. For a more accurate comparison, we performed real time-PCR with two internal reference microRNAs: miR-103 and miR-191 (Fig. 3). For miR-132, we found a 3.3-fold increase compared to controls (*p* = 0.033), when the signals were normalized to miR-103 (data not shown) and a 2.3-fold increase compared to controls (*p* = 0.036), when the signals were normalized to miR-191 (Fig. 3A). For miR-34a, we found a 4-fold increase compared to controls (*p* = 0.0046), when the signals were normalized to miR-103 (data not shown) and the same increase (4-fold; *p* = 0.001), when signals were normalized to miR-191 (Fig. 3B).

### In PBC, Keap1 protein levels are elevated, mRNA levels are diminished, and Keap1 is present in different cellular compartments

**Figure 4:**
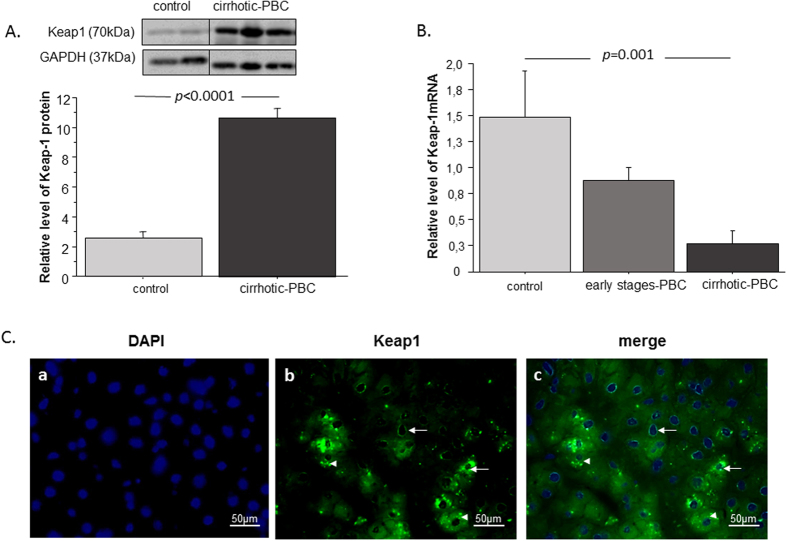
The hepatic expression of Keap1 in liver tissues of patients with PBC and controls. (**A**) Keap1 protein levels were determined with densitometry analyses, after normalization to GAPDH as a loading control. (**B**) Keap1 mRNA levels were estimated in patients with cirrhotic PBC, patients with early stage PBC, and controls. Results were normalized to 18sRNA. Bars indicate the mean ± SEM. (**C**) Representative immunofluorescence micrographs show liver sections from patients with PBC. (a) Nuclei are stained with DAPI (blue). (b) Immunofluorescence staining of Keap1 (green) shows its abundance in hepatocytes. (c) Arrows indicate the perinuclear and nuclear localizations of Keap1whereas arrowheads indicate cytoplasmic localization of Keap1.

Keap1 is a ubiquitin ligase that orchestrates the Nrf2 pathway in cells. Therefore, to determine why Nrf2 protein levels were elevated in PBC, we estimated the levels of Keap1 protein and mRNA (Fig. 4). We found that Keap1 protein levels were significantly elevated in patients with PBC compared to controls (4.2-fold, p < 0.0001; Fig. 4A). However, Keap1 mRNA expression was downregulated in cirrhotic PBC compared to controls and early-stage PBC (5.5-fold, p = 0.015; 2.8-fold, p = 0.014; Fig. 4B). Next, we analysed Keap1 localization with immunofluorescence (Fig. 4C). We found that the Keap1 protein was present in different cellular compartments. Immunofluorescence and DAPI staining showed that Keap1 was present in both the perinuclear and nuclear regions of liver cells (Fig. 4C.c).

### In PBC, the localization of both Keap1 and Nrf2 in liver tissue is different from control tissue

**Figure 5:**
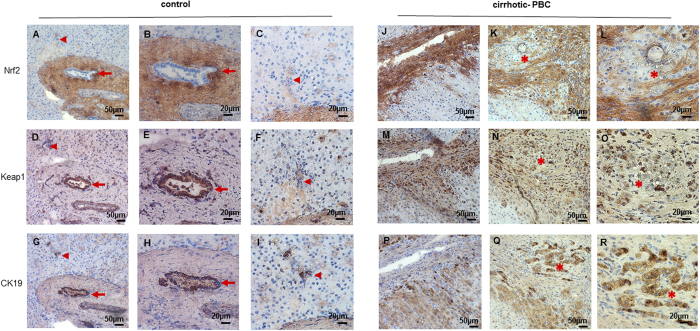
Liver expression of Nrf2, Keap1 and CK19 proteins in patients with cirrhotic PBC and controls. Representative immunohistochemical staining of Nrf2 (**A,B,C,J,K,L**), Keap1 (**D,E,F,M,N,O**) and CK19 (**G,H,I,P,Q,R**) proteins in serial sections of liver tissue from healthy controls **(A–I)** and cirrhotic PBC **(J–R)**. In healthy tissue, CK19-positive cells are marked by arrow (large bile duct) or arrowhead (small bile duct). In sections of cirrhotic livers, the corresponding areas are labelled by asterisks. Nrf2 was present only in fibrotic areas **(J,K,L),** in contrast to Keap1 which was expressed in fibrotic areas as well as in nodules **(M,N,O).** Original magnification 200x or 400x.

The analysis of consecutive liver slices revealed the localization of Keap1 and Nrf2 in PBC and control tissues. Immunostaining for biliary marker, cytokeratin 19 (CK19) allows to visualize biliary epithelial cells (Fig. 5G,H,I,P,Q,R). In healthy tissue, Nrf2 was expressed neither in large bile ducts (Fig. 5A,B, arrow), nor in small bile ducts (Fig. 5A,C; arrowhead), while Keap1 was present in large bile duct (Fig. 5D,E; arrow) but not in small bile duct (Fig. 5D,F; arrowhead). In PBC CK19-positive cells formed spike-like extensions oriented perpendicular to the nodular perimeter (Fig. 5P,Q,R) and did not express Nrf2 (Fig. 5K,L; asterisk). Nrf2 was present only in fibrotic areas (Fig. 5J,K,L), in contrast to Keap1 which was expressed in fibrotic areas as well as in nodules (Fig. 5M,N,O).

### The p62 protein level is upregulated in PBC

**Figure 6:**
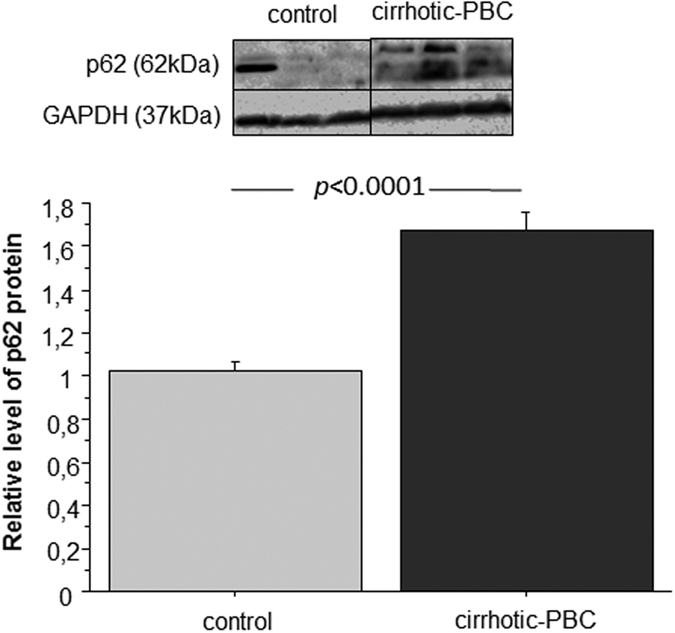
Expression of p62 protein in liver tissues derived from patients with PBC and controls. Changes in p62 protein levels were determined with densitometry analyses after normalization to GAPDH as a loading control. Bars indicate the mean ± SEM.

p62 degrades Keap1 in an autophagy-dependent manner. However, when autophagy is inhibited, both proteins accumulate in the cell without fulfilling their function. Thus, to shed light on the increased levels of Keap1 in PBC, we checked the p62 protein levels in liver extracts with Western blot analyses (Fig. 6). The results showed that p62 was significantly upregulated in PBC compared to controls (1.6-fold, p < 0.0001).

## Discussion

Despite data that confirmed oxidative stress generation during PBC progression, there is a lack of knowledge on how liver cells protect against the deterioration of redox homeostasis in PBC. The Nrf2 transcription factor is a known master regulator of antioxidant defence in cells; it works by orchestrating the induction of various cytoprotective genes.

In normal conditions, Nrf2 exhibits a short half-life of approximately 20 min; however, its half-life is extended upon disruption of the interaction between Nrf2 and its potent repressor, Keap1^26^. Thus, in PBC an oxidative stress should, on one hand, stabilize the Nrf2 protein, and on the other hand, induce *NRF2* gene expression. Consequently, PBC should be associated with elevated levels of Nrf2 protein. In liver tissues of patients with PBC, we showed that the level of Nrf2 protein was indeed elevated compared to control tissues. However, we observed the opposite result for *NRF2* gene expression. We found that Nrf2 mRNA levels were 3.5-fold lower in cirrhotic PBC than in control and non-cirrhotic PBC conditions. Importantly, compared to controls, *NRF2* gene expression was not elevated in tissues from patients in the early stages of PBC. However, it is worth mentioning that the circulating redox balance is also impaired in patients with early stages of PBC^12^. Thus, the response to oxidative stress should be induced, particularly at the beginning of disease progression, when liver tissues have not undergone pathological changes to the extent observed in cirrhotic tissues. Additionally immunohistochemical analysis of liver tissue from PBC patients revealed the presence of Nrf2 mainly in fibrotic areas and not in biliary epithelial cells or in hepatocytes.

The lack of *NRF2* gene induction combined with the increased Nrf2 protein levels in PBC suggested that Nrf2 protein degradation was inhibited, and accordingly, Nrf2 accumulated. Of note, it has been documented that, upon Nrf2 translocation into the nucleus, it can activate its own gene expression^27^. Taking these data into consideration, the question arises as to whether Nrf2 is active as a transcription factor in pathologically altered liver tissue. In addition, the Nrf2/Keap1 pathway tightly controls the expression of genes that encode the antioxidant enzymes, HO-1 and GCLC. Previous studies have reported that HO-1 can reduce oxidative injury, attenuate the inflammatory response, inhibit cell apoptosis, and maintain cellular homeostasis. The beneficial effects of HO-1 on liver function has been confirmed in studies on acute liver failure, alcoholic or viral hepatitis, chronic inflammation, fibrosis, and cirrhosis^16^,^28^. GCLC is involved in glutathione metabolism. Glutathione is an integral constituent in normal liver function; it provides critical defence against oxidative cell damage and it provides an important osmotic force, which drives the flow of bile^29^,^30^,^31^. Our results have shown that the levels of HO-1 and GCLC proteins were diminished in liver extracts of patients with PBC. Our quantitative data on *HO-1* gene expression levels revealed a concomitant decrease in the amount of HO-1 mRNA in PBC compared to controls. Of note, we observed low HO-1 mRNA levels in patients, in both the cirrhotic stage and in the early stages of the disease. This observation confirmed that protection against oxidative stress was impaired at the outset of the disease; thus, this impairment may lead to PBC progression and liver failure. In summary, our data suggested that reductions in Nrf2-induced HO-1 and GGCT proteins, combined with a lack of induction of the *NRF2* gene in PBC might result from the impaired function of Nrf2 protein in PBC, and the inhibited recovery of the Nrf2 protein pool.

In search of another explanation for the reduced levels of Nrf2 mRNA in PBC, we decided to focus on epigenetic mechanisms. Messenger RNA levels may be regulated by small, non-coding microRNAs that target specific genes. To date, a few studies have investigated the role of microRNAs in PBC pathology^32^,^33^,^34^. However, the relationship between antioxidant defence and microRNA expression in PBC remains unknown. Several studies have shown that Nrf2 was directly targeted by numerous microRNAs^35^,^36^,^37^. Microarray studies on microRNA expression profiles and recent studies on Nrf2-specific microRNAs have shown that miR-132 16 and miR-34a^38^ could alter Nrf2 mRNA expression. Our results have shown that, in PBC, the induction of microRNAs may contribute to reduction in Nrf2 mRNA levels, which led to impaired cellular defence against oxidative stress.

In PBC, low Nrf2 mRNA levels appear to result from alterations in both Nrf2 transcription and epigenetic regulation of *NRF2* gene expression; however, the cause of high Nrf2 protein levels remains unclear. The cellular function of Nrf2 is tightly controlled by Keap1. Under basal conditions, Keap1 binds to Nrf2, which results in either the proteasome-mediated degradation of Nrf2 or the sequestration of Nrf2 in the cytoplasm. Oxidative stress inactivates Keap1, which leads to the stabilization and nuclear translocation of Nrf2. Thus, Keap1 activity is negatively correlated with Nrf2 activation. To extend the complexity of our understanding of Nrf2/Keap1 signalling, we focused on Keap1 as the second important player of the antioxidant response. Surprisingly, we found that the levels of Keap1 protein were elevated in liver extracts from patients with PBC compared to extracts from controls. Additionally, an immunofluorescence analysis revealed the cellular localization of Keap1. In PBC, this protein was omnipresent in different cellular compartments, including the nuclear and perinuclear regions and in different cellular types. Previous studies have reported that the amino acid sequence of Keap1 contained a nuclear export signal (NES). Thus, via NES, Keap1 provides nuclear export for Nrf2, which attenuates the Nrf2-mediated antioxidant response; this activity constitutes the postinduction repression of Nrf2^39^,^40^. Those observations might partially explain the low HO-1 and GCLC levels in PBC, despite the increased Nrf2 protein levels. To gain a wider understanding, we estimated *KEAP1* gene expression in PBC. Interestingly, KEAP1 mRNA was downregulated in patients with cirrhotic PBC compared to controls; we also observed diminished KEAP1 mRNA levels in patients in the early stages of PBC, however these results were not statistically significant.

The increased Keap1 protein levels in PBC, despite the reduction in KEAP1 mRNA might be explained by the inhibition of cellular Keap1 degradation, followed by its accumulation in different cellular compartments. Importantly, the cellular accumulation of proteins is often considered a hallmark of pathology, which can be manifested by impairments in protein function and by inhibition of gene expression via a negative feedback loop. In the liver, Keap1 is constitutively degraded by autophagy in a p62- dependent manner. Thus, in seeking an explanation for the increased levels of Keap1 in PBC, we considered the crosstalk between the Nrf2/Keap1 and autophagy pathways^17^,^41^,^42^,^43^,^44^. This assumption was justified by our observations of elevated miR-34a levels in PBC. Like Nrf2, mir-34a influences the transcription of numerous proteins involved in autophagy, including ATG4B, Beclin-1, LC3B II/I, and Sirtuin-1^45^,^46^,^47^. Moreover, it was shown that aberrant accumulation of p62 in autophagy-deficient mice disrupted the Nrf2-Keap1 interaction, which led to Nrf2 accumulation and liver damage^48^. Furthermore, damaged cholangiocytes in PBC were characterized by p62 aggregation^49^,^50^,^51^. In accordance with the discussed data, we showed that the levels of p62 were increased in livers explanted from patients with PBC, compared to explants from controls; this finding was consistent with the increase in Keap1 protein levels. Of note, p62 gene is activated by Nrf2. However, in light of our data and data from other groups, we postulate that the high p62 levels observed in PBC were related to p62 accumulation, rather than its gene induction by Nrf2^17^,^52^. The accumulation of p62 was a sign of aberrant autophagy, which caused insufficient processing of the damaged proteins that were bound to p62, such as Keap1. These results might explain the observed high levels of both Keap1 and p62 proteins in PBC and the simultaneous decrease in *KEAP1* gene expression.

In summary, the present study revealed that PBC was associated with an impairment in the liver response to oxidative damage. Despite continuous oxidative damage to liver tissues, we observed downregulated gene expression of Nrf2, a key regulator of redox homeostasis. In pathologically altered liver, two microRNAs that inhibit Nrf2 expression were overexpressed. Moreover, Keap1, which fine-tunes Nrf2 activity, appeared to accumulate with p62 in cells, but it lacked the ability to fulfil its function. Our results on miR-34a overexpression and hepatic p62 accumulation in PBC have provided new insight, because they represent a link between autophagy regulation in PBC and microRNA expression in diseased liver. Thus, the lack of an appropriate response to oxidative stress could be an additional factor that contributes to liver failure in PBC.

## Materials and Methods

### Liver tissue specimens

This study included patients diagnosed with PBC, based on criteria established by the European Association for the Study of the Liver. Non-cirrhotic liver species were obtained by percutaneous needle liver biopsy (2–3 mm^2^, immersed in *RNAlater* solution) from PBC patients who underwent liver biopsy for histological assessment (early-stages PBC). Samples of cirrhotic liver tissue were collected from PBC patients with histologically proven cirrhosis (cirrhotic PBC) who underwent liver transplantation (volume of sample: ∼1 cm^3^, immediately frozen). Control liver tissues with no microscopic changes of liver disease identified by a pathologist, were secured from large margin liver resections of colorectal metastases as previously described (controls)^53^. Frozen liver samples were stored at −80 °C in the Department of Medical Biology, Pomeranian Medical University, Szczecin.

### RNA extraction and quantification of gene expression

Total RNA was isolated from the livers of patients with cirrhotic PBC (n = 17), patients with early-stage PBC (n = 22), and controls (n = 15), with the RNeasy Mini kit (Qiagen), according to the manufacturer’s protocol. cDNA synthesis was carried out with the SuperscriptTM II RT kit (Invitrogen), according to the protocol previously described^54^; these cDNA samples were stored at −20 °C. We performed quantitative real-time PCR to measure the expression of specific target genes with commercially available Gene Expression Assays and a 7500 Fast Real-Time PCR System (Applied Biosystems). We measured the following transcripts in this study: HO-1, Keap-1, Nrf2, and control human 18sRNA. Briefly, each assay comprised a 20-μl reaction mixture that contained 10 μl TaqMan^®^ Gene Expression PCR Master Mix (Applied Biosystems), 2 μl diluted cDNA template, and 1 μl of the probe/primer assay mix. Fluorescence data were analysed with 7500 Software v2.0.2. (Applied Biosystems). The expression of target genes was calculated with the ΔΔCt method of relative quantification.

### Protein expression analysis

Proteins from frozen cirrhotic PBC liver tissues (n = 24) and controls (n = 16) were extracted with homogenization in an ice-cold RIPA buffer (50 mM Tris-HCl pH = 8, 150 mM NaCl, 1% NP-40, 0.5% NaDOC, 0.1% SDS, 1 mM EDTA, 100 mM PMSF, 100 mM NaF), which contained a protease inhibitor cocktail and PhosSTOP (Roche Diagnostics GmbH). Proteins were quantified with the bicinchoninic acid assay (Micro BCA™ Protein Assay Kit; Thermo Scientific). Protein extracts (100 μg) from each liver sample were electrophoresed on SDS polyacrylamide gels, and subsequently, the separated proteins were blotted onto PVDF membranes (Thermo Scientific) under semi-dry transfer conditions. Membranes were blocked for 1 h at room temperature with TBST, which contained 5% (w/v) milk (Merck). The membranes were probed with the following primary antibodies: anti-GCLC (Thermo Scientific, #PA5–16581; 1:1000 dilution), anti-Keap1 (Santa Cruz, #33569; 1:1000 dilution), anti-p62 (R&D, #MAB8028; 1:250 dilution), anti-Nrf2 (Cell Signalling, #12721; 1:200 dilution), and anti-GAPDH (Santa Cruz, #sc25778; 1:5000 dilution, or sc-365062; 1:5000 dilution). For the detection of antigen-antibody complexes, we employed a peroxidase-conjugated anti-rabbit secondary antibody (NA9340V, Amersham, GE Healthcare, 1:5000 dilution) or an anti-mouse secondary antibody (Jackson ImmunoResearch, #115-035-146; 1:50 000 dilution). Protein expression was detected with an enhanced chemiluminescence detection system (Chemiluminescent HRP Substrate, Millipore). Bands were visualized and quantified with the MicroChemi 2.0 System and GelQuant software (Israel).

### ELISA

To estimate the concentration of HO-1 in liver tissues, we performed the ELISA test (Enzo Life Sciences, #ADI-EKS-800), according to the manufacturer’s protocol. Proteins from frozen cirrhotic PBC liver tissues and control frozen liver tissues were extracted with homogenization in the appropriate ice-cold buffer, which contained a protease inhibitor cocktail (Roche Diagnostics GmbH). We quantified proteins with the bicinchoninic acid assay (Micro BCA™ Protein Assay Kit; Thermo Scientific).

### Immunohistochemistry and immunofluorescence

Frozen liver sections (6 μm) derived from patients with PBC and controls were fixed in a methanol and acetone mixture (1:1) at −20 °C for 5 min. The cut order of sections was known and described. We examined the proteins of interest with immunohistochemistry analyses. Briefly, sections were treated with Avidin/Biotin Blocking Kit (#SP-2001, Vector Laboratories) and next incubated with 3% H202 diluted in methanol. Then sections were probed with rabbit anti-Keap1 (Santa Cruz, #33569; 1:50 dilution), anti-Nrf2 (Cell Signaling, # 12721 S; 1:20 dilution), anti-CK19 (Santa Cruz, #33119; 1:50 dilution). Then, sections were incubated with either biotinylated anti-mouse/anti-rabbit IgG (#BA-1400, Vector Laboratories) or biotinylated anti-goat IgG (#BA-9500, Vector Laboratories). Reactions were visualized using ABC Vectastain and DAB kits (DAKO, Agilent Technologies, Denmark). The negative controls, in which the primary antibodies were omitted, were included in the study and uniformly demonstrated no reaction. For immunofluorescence sections were probed with rabbit anti-Keap1 (Santa Cruz, #33569; 1:500 dilution). Then, sections were incubated with fluorescein-isothiocyanate (FITC)-conjugated anti-rabbit IgG (#711-095-152, Jackson ImmunoResearch; 1:500 dilution). Sections were stained with 4’,6diamidino-2-phenylindole (DAPI; H-1200, VECTOR) to visualize cell nuclei. Then, sections were mounted with Vectrashield Mounting Medium. Negative controls were prepared by omitting the primary antibodies (data not shown). Images were acquired with a ZEISS Axio Imager Z2 fluorescence microscope, equipped with the Zen Pro 2011 acquisition program.

### MicroRNA Assay

Total RNA was isolated from samples of livers explanted from patients diagnosed with PBC (n = 4) and from liver samples from age- and gender-matched control donors (n = 4), with the miRNeasy Mini Kit (Qiagen). Microarray analysis was performed with Affymetrix GeneChip miRNA 4.0 arrays by the Microarray Core facility at Boston University (http://www.bumc.bu.edu/microarray/). The false discovery rate (FDR) correction was applied to p-values (a.k.a. q-values) to determine the significance of a given test, when many hypotheses (e.g., ~2,600 microRNAs) were tested at once. The results were confirmed with real-time PCR on PBC liver samples (n = 15) and control liver samples (n = 10). Briefly, cDNA was synthesized with the *Applied Biosystems* TaqMan *Advanced miRNA* cDNA Synthesis Kit, according to the manufacturer’s protocol. We measured the expression of miR-132, miR-34a, miR-103, and miR191 with commercially available TaqMan^®^ Advanced miRNA Assays (Applied Biosystems). Then, each assay comprised a 20-μl reaction mixture that contained 10 μl TaqMan^®^ Fast Advanced Master Mix (Applied Biosystems), 5 μl diluted cDNA template, and 1 μl of the probe/primer assay mix. Fluorescence data were analysed with 7500 Software v2.0.2. (Applied Biosystems). The expression of target genes was calculated with the ΔΔCt method of relative quantification.

### Ethics

Written informed consent was obtained from each patient included in the study. The study protocol was approved by the Ethics Committee of Pomeranian Medical University and conforms to the ethical guidelines of the 1975 Declaration of Helsinki (6th revision, 2008).

## Additional Information

**How to cite this article**: Wasik, U. *et al*. Protection against oxidative stress mediated by the Nrf2/Keap1 axis is impaired in primary biliary cholangitis. *Sci. Rep.* **7**, 44769; doi: 10.1038/srep44769 (2017).

**Publisher's note:** Springer Nature remains neutral with regard to jurisdictional claims in published maps and institutional affiliations.
